# Nanopriming with Multi-Walled Carbon Nanotubes Enhances Abiotic Stress Tolerance in Sunflower Seeds

**DOI:** 10.3390/plants15040584

**Published:** 2026-02-12

**Authors:** Thalita Maciel Pereira, Antonio Rodriguês da Cunha Neto, Leticia de Aguila Moreno, Juliano Elvis de Oliveira, Marcelo Pedrosa Gomes, Fernanda Carlota Nery, Everson Reis Carvalho, Michele Valquíria dos Reis

**Affiliations:** 1 Department of Agriculture, Federal University of Lavras, Lavras CEP 37203-202, Minas Gerais, Brazil; thalitatmp15@gmail.com (T.M.P.); juliano.oliveira@ufla.br (J.E.d.O.); eversoncarvalho@ufla.br (E.R.C.); 2 Department of Biotechnology, Federal University of Alfenas, Alfenas CEP 37130-001, Minas Gerais, Brazil; antoniorodrigues.biologia@gmail.com; 3 Department of Botany, Federal University of Parana, Curitiba CEP 81531-970, Paraná, Brazil; marcelo.gomes@ufpr.br; 4 Department of Biotechnology, Federal University of São João del Rei, São João del Rei CEP 36307-352, Minas Gerais, Brazil; fernandacarlota@ufsj.edu.br

**Keywords:** nanotechnology, nanomaterials, physiological seed quality, propagation

## Abstract

Sunflower (*Helianthus annuus* L.) is a crop with ornamental and energetic potential, but its propagation is challenged by abiotic stresses such as salinity and water deficit. Ensuring high-quality propagation materials is crucial for healthy plant development. Nanotechnology offers innovative tools to enhance seed performance, stress tolerance, and production efficiency. Among these, carbon nanotubes, a strong, conductive, and thermally resistant material, have shown promise in improving seed quality. This study aimed to evaluate the effects of nanopriming with multi-walled carbon nanotubes (MWCNTs) on the physiological and biochemical performance of sunflower seeds under accelerated aging and stress conditions. Seeds were treated with 100, 200, or 400 mg·L^−1^ MWCNTs, and parameters such as germination percentage, seedling growth, pigment profile, and oxidative stress indicators were analyzed. The 200 mg·L^−1^ concentration enhanced germination, root and shoot development, and antioxidant enzyme modulation, while the 400 mg·L^−1^ dose increased reactive oxygen species, indicating toxicity. Under saline and drought-like conditions, nanopriming with 100–200 mg·L^−1^ mitigated oxidative damage more effectively than hydropriming. MWCNTs also influenced pigment synthesis, affecting chlorophyll and carotenoid levels. These findings support the potential of carbon nanotube-based nanopriming to improve seed vigor and stress tolerance in sunflower cultivation, though further environmental safety assessments are required.

## 1. Introduction

Sunflower (*Helianthus annuus* L.) is a crop with broad applicability, notable for its production of grains and vegetable oil, ornamental value, use in animal feed, phytoremediation of soils, and green manure. It is among the plant species with the greatest potential for renewable energy research [1,2]. Due to its sensitivity to extreme temperatures and drought, advanced propagation methods and high-quality starter seeds can help overcome these stresses, aiding in achieving high productivity [3].

Seed germination is a process in which physiological and morphological changes occur in an orderly sequence, depending on a set of environmental conditions. It can be influenced by factors such as water deficiency, adverse temperatures, and saline soils [4]. These stressors can induce oxidative stress in seeds [5], negatively affecting seedling growth and plant productivity. Therefore, the use of seed treatments that promote rapid and uniform seedling establishment, even under stressful conditions, are essential for achieving high sunflower yields.

One of the most widely used techniques to standardize the germination process is physiological conditioning or priming, which involves hydrating the seed to a level sufficient to allow pre-germinative metabolic events, yet insufficient for radicle protrusion. To most common methods used to achieve this stagecare hydropriming and osmopriming [5]. Current advances in seed priming represent promising approaches for enhancing seed germination and plant development under various environmental conditions [6]. In this context, the application of nanoparticles through seed priming, known as nanopriming, is an innovative research area, with promising preliminary results. Recent studies have shown that nanopriming surpasses traditional priming due to its ability to activate antioxidant enzymes, open nanopores in the seed coat, and stimulate stress signaling pathways [7]. The mechanisms underlying nanopriming result in increased germination and vigor under various environmental conditions [8].

In nanotechnology, a particle is defined as a piece of matter with defined physical boundaries [9]. At the nanoscale, materials tend to exhibit altered properties, allowing the creation or enhancement of specific products. These benefits include controlled release, stability, and targeted delivery of bioactive molecules, such as hormones, nutrients, pharmaceuticals, and antioxidants, to specific sites of action [10]. Nanotechnology plays a promising role in transforming food production and agriculture by reducing excessive reliance on fertilizers and chemical pesticides, while also enhancing seed germination and vigor [11]. Beyond agricultural productivity, nanomaterials are increasingly being integrated into plant-based environmental remediation strategies. This approach, termed nanophytoremediation, combines the efficiency of nanoparticles with plant resilience, enhancing contaminant uptake and degradation while mitigating stress effect on plants [12].

Carbon nanotubes are carbon-based materials that exhibit promising technological applications owing to their strength, flexibility, and high conductivity [13]. They can be visualized as rolled-up sheets of graphene-forming cylinders, and possess a unique combination of stiffness and strength, high surface-area-to-volume ratio, chemical stability, cellular penetration capacity, and potential for functionalization, which enhances their interaction with plant tissues and the efficient transport of molecules. The use of multi-walled carbon nanotubes (MWCNTs) on seeds offers specific advantages, such as greater structural robustness and stability compared to single-walled carbon nanotubes (SWCNTs), enabling more efficient interaction with seeds during nanopriming [14]. Additionally, due to their multilayered structure, MWCNTs demonstrate higher efficiency in transporting bioactive molecules during nanopriming compared to metal-based nanoparticles, while exhibiting a reduced potential for toxicity [15]. Furthermore, MWCNTs help plants cope with stress by increasing antioxidant activity during early stages of development [16]. Finally, MWCNTs are typically more affordable and easier to synthesize at scale compared to the other materials, making them more viable for large-scale agricultural applications [17].

These properties support their application in agriculture, enabling improved physiological efficiency of seeds and potential performance gains under adverse conditions [18]. Therefore, this study aimed to investigate the physiological, morphological, and biochemical responses of sunflower seeds exposed to multi-walled carbon nanotubes through nanopriming with carbon nanotubes. It was hypothesized that nanopriming with multi-walled carbon nanotubes (MWCNTs) enhances the germination performance and improves the physiological and biochemical stress tolerance of sunflower seeds under abiotic stress conditions, compared to both hydropriming and untreated seeds.

## 2. Results

### 2.1. Accelerated Aging Experiment

#### 2.1.1. Physiological Seed Parameters

**Figure 1:**
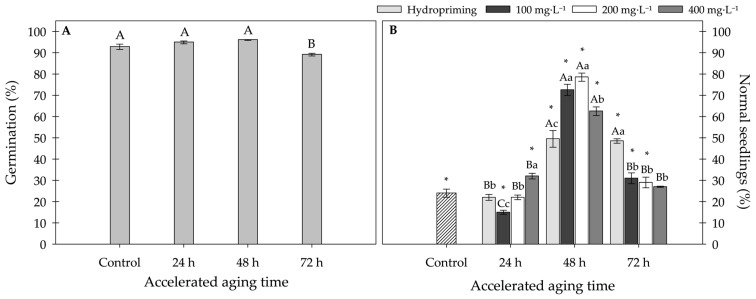
(**A**) Germination (%) of sunflower seeds at different accelerated aging times. (**B**) Normal sunflower seedlings (%) exposed to different concentrations of carbon nanotubes under different accelerated aging times. Error bars represent the standard error (SE). Equal letters do not differ statistically from each other according to the Scott-Knott test (*p* ≥ 0.05), uppercase letters compare accelerated aging times within the same priming treatment and lowercase letters compare conditioning with nanotubes within the accelerated aging time. Symbol “*” indicates a statistical difference from the control (without accelerated aging and priming treatment) by the Dunnett test (*p* ≥ 0.05).

The results showed that there was no significant interaction between accelerated aging and physiological conditioning with MWCNTs (multi-walled carbon nanotubes) on sunflower seed germination. For this parameter, only accelerated aging showed a statistically significant difference, with the 72 h treatment being inferior to the others, achieving 89% seed germination. The other treatments (0, 24, and 48 h) did not differ significantly from each other, with an average of 95% sunflower seed germination (Figure 1A).

In contrast to what was observed for germination percentage, the interaction between priming treatments was significant for the percentage of normal seedlings. When comparing seeds from the control group (without accelerated aging and priming treatment) with the other treatments, a higher percentage of normal seedlings was observed in seeds exposed to accelerated aging for 48 h. Under this condition, seeds treated with 100 mg·L^−1^ and 200 mg·L^−1^ concentrations of MWCNTs showed superior performance compared to the other treatments, indicating a positive effect of carbon nanotube application.

Furthermore, it is noteworthy that the percentage of normal seedlings after hydropriming and accelerated aging for 48 and 72 h did not differ significantly from each other and was higher than that observed in the control group and seeds subjected to 24 h of accelerated aging. However, under the 72 h accelerated aging condition, unlike what was observed at 48 h, the presence of MWCNTs reduced the percentage of normal seedlings (Figure 1B).

Based on previous results, the optimal condition for accelerated aging of sunflower seeds were determined to be 48 h, according to the parameters of normal seedling development. Therefore, subsequent evaluations focused on comparing the different concentrations of carbon nanotubes within the 48 h period.

#### 2.1.2. Morphological Seedling Parameters

**Figure 2:**
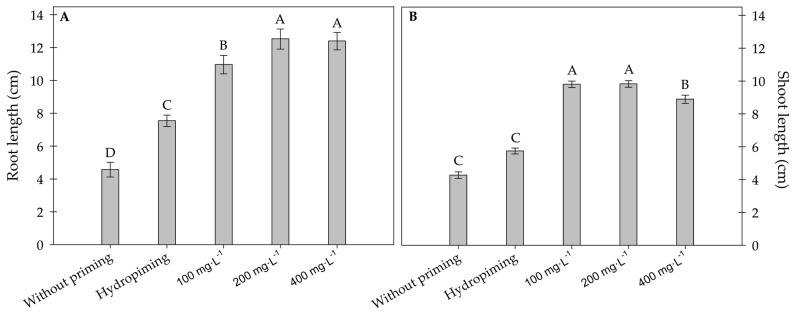
(**A**) Root and (**B**) shoot lengths of sunflower seedlings exposed to different concentrations of carbon nanotubes after 48 h of accelerated aging. Error bars represent the standard error (SE). Equal letters do not differ statistically from each other according to the Scott-Knott test (*p* ≥ 0.05).

It was observed that treatments with 200 mg·L^−1^ and 400 mg·L^−1^ resulted in the greatest root development, followed by the 100 mg·L^−1^ concentration. Hydropriming produced lower values than the treatments with carbon nanotubes, but higher than that of the seeds without conditioning (Figure 2A). On the other hand, regarding shoot length (Figure 2B), the carbon nanotube concentrations of 100 mg·L^−1^ and 200 mg·L^−1^ yielded the best results, followed by the 400 mg·L^−1^ treatment.

**Figure 3:**
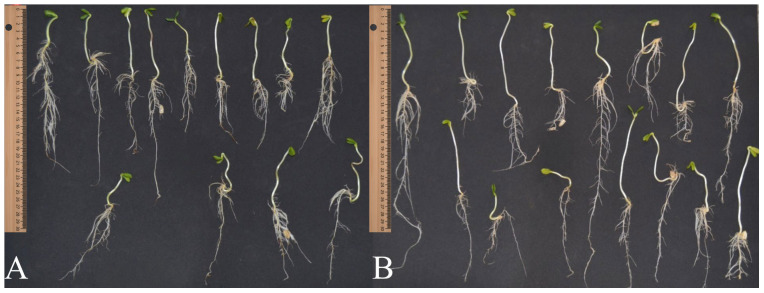
Comparison between sunflower (**A**) control treatment (seedlings not exposed to carbon nanotubes) and (**B**) seedlings exposed to 200 mg·L^−1^ of carbon nanotubes after the same accelerated aging condition (48 h) (n = 15 seedlings).

Both the hydropriming and unconditioned treatments exhibited the shortest shoot lengths compared to those treated with carbon nanotubes. The differences in shoot and root sizes between two treatments (200 mg·L^−1^ MWCNTs and the control without MWCNTs) can be observed in Figure 3A,B.

As illustrated in Figure 3A,B, seedlings treated with 200 mg·L^−1^ of MWCNTs showed visually superior shoot and root development compared to the control.

#### 2.1.3. Colorimetric Parameters of Cotyledonary Leaves

**Figure 4:**
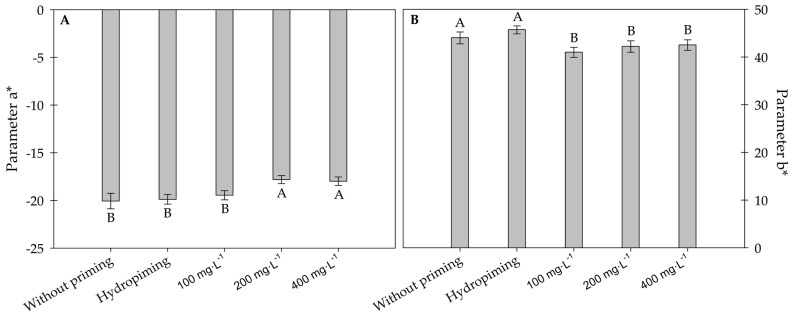
Parameters a* (**A**) and b* (**B**) of sunflower seedlings exposed to different concentrations of carbon nanotubes after 48 h of accelerated aging. Error bars represent the standard error (SE). Equal letters do not differ statistically from each other according to the Scott-Knott test (*p* ≥ 0.05).

When analyzing the colorimetric parameters of sunflower seedlings exposed to carbon nanotubes, no significant difference was observed in the L* parameter, which refers to the brightness of the sample. Thus, regardless of treatment, the sunflower seedlings had a value of 48.34 (dimensionless) for this parameter. For the a* parameter, the values obtained were negative, indicating that the samples were located within the green spectrum. Among the treatments, the highest a* values were observed in seedlings treated with 200 and 400 mg·L^−1^ (Figure 4A).

Regarding the b* parameter, the values obtained were positive, indicating a placement within the yellow spectrum. For this parameter, carbon nanotube treatments, regardless of concentration, resulted in lower b* values compared to both the hydropriming technique and those without priming (Figure 4B). This reduction indicated a slight shift in the yellow tone toward the blue spectrum which may be associated with changes in the biochemical composition or physiological state of the seedling.

#### 2.1.4. Quantification of Hydrogen Peroxide and Lipid Peroxidation

**Figure 5:**
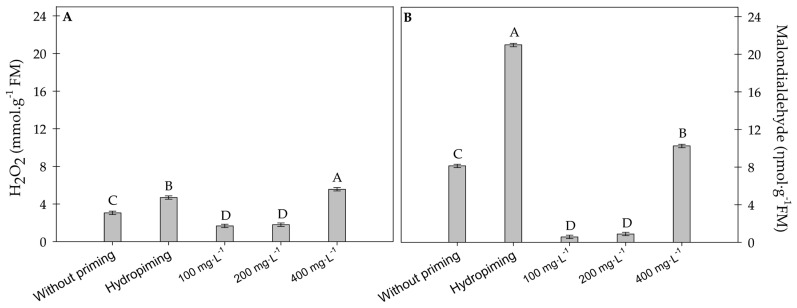
(**A**) Hydrogen peroxide and (**B**) lipid peroxidation in sunflower seedlings exposed to different concentrations of carbon nanotubes after 48 h of accelerated aging. Error bars represent the standard error (SE). Equal letters do not differ statistically from each other according to the Scott-Knott test (*p* ≥ 0.05).

Seedlings exposed to physiological conditioning with 100 and 200 mg·L^−1^ of carbon nanotubes produced lower levels of hydrogen peroxide (H_2_O_2_) compared to the other treatments. At the highest concentration tested, 400 mg·L^−1^ of carbon nanotubes, there was an increased expression of H_2_O_2_, indicating that elevated concentrations promote the production of ROS (Figure 5A). A similar trend was observed for lipid peroxidation, with the best results observed in treatments with 100 and 200 mg·L^−1^ of carbon nanotubes (Figure 5B).

#### 2.1.5. Quantification of Antioxidant System Enzymes

**Figure 6:**
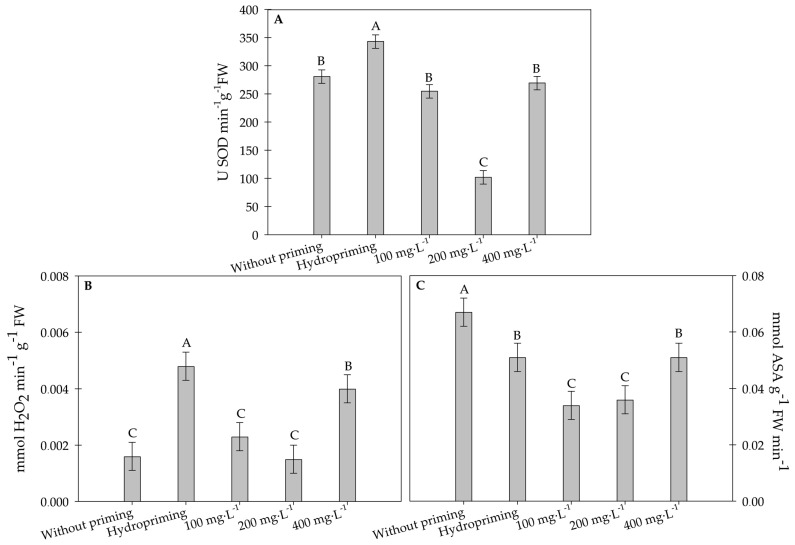
(**A**) Superoxide dismutase (SOD), (**B**) catalase (CAT), and (**C**) ascorbate peroxidase (APX) activities in sunflower seedlings exposed to different concentrations of carbon nanotubes after 48 h of accelerated aging. ASA stands for ascorbic acid. Error bars represent the standard error (SE). Equal letters do not differ statistically from each other according to the Scott-Knott test (*p* ≥ 0.05).

The hydropriming treatment exhibited the highest activity of superoxide dismutase (SOD), which was significantly greater than that of the other treatments (Figure 6A). Treatments with 100 mg·L^−1^ and 400 mg·L^−1^ of carbon nanotubes also resulted in significantly high SOD activities, though not statistically different from the control seeds. In contrast, the treatment with 200 mg·L^−1^ of carbon nanotubes showed the lowest SOD activity, significantly lower than the other treatments.

Regarding catalase (CAT), the treatment with 400 mg.L^−1^ of carbon nanotubes also exhibited relatively high activity but was significantly lower than hydropriming (Figure 6B). Treatments without physiological conditioning, as well as those with 100 mg L^−1^ and 200 mg·L^−1^ of carbon nanotubes, showed the lowest CAT activities, with no significant differences between them, and all were lower than the hydropriming treatments.

The treatment without priming showed the highest ascorbate peroxidase (APX) activity, which was significantly greater than that of all the other treatments (Figure 6C). Treatments with 100 mg·L^−1^ and 200 mg·L^−1^ of carbon nanotubes exhibited the lowest APX activities, with no significant differences between them. The treatment with 400 mg·L^−1^ of carbon nanotubes resulted in significantly higher APX activity compared to the 100 mg·L^−1^ and 200 mg·L^−1^ treatments, but similar to hydropriming and lower than the non-primed treatment.

### 2.2. Germination Stressful Conditions Experiment

#### Physiological Seed Parameters

**Figure 7:**
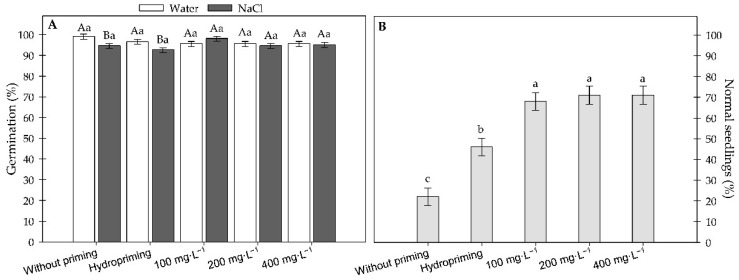
(**A**) Germination (%) of sunflower seeds under water and salinity (NaCl) across different concentrations of carbon nanotubes; (**B**) normal sunflower seedlings (%) exposed to different concentrations of carbon nanotubes. Error bars represent the standard error (SE). Equal letters do not differ statistically from each other according to the Scott-Knott test (*p* ≥ 0.05). Uppercase letters compare germination conditions within the same carbon concentration, and lowercase letters compare the different carbon concentrations within the same germination condition.

There was no significant interaction between the concentrations and the germination conditions. For the results obtained from the second batch of sunflower seeds, Figure 7A shows that treatments with hydropriming and MWCNTs did not significantly differ in germination rates, regardless of water or NaCl conditions. However, in treatments without priming or hydropriming alone, germination was lower in the presence of NaCl (−0.45 MPa). In contrast, at MWCNT concentrations of 100, 200, and 400 mg·L^−1^, no significant differences were observed between the two solutions, suggesting that nanopriming mitigated the effects of salt stress at least during the initial germination phase.

Figure 7B shows the significant differences among treatments in the percentage of normal seedlings under salt stress. The no-priming treatment resulted in the lowest rate, whereas hydropriming led to a significant increase. Treatments with 100, 200, and 400 mg·L^−1^ of MWCNTs showed the highest percentages, with no significant differences among them.

**Figure 8:**
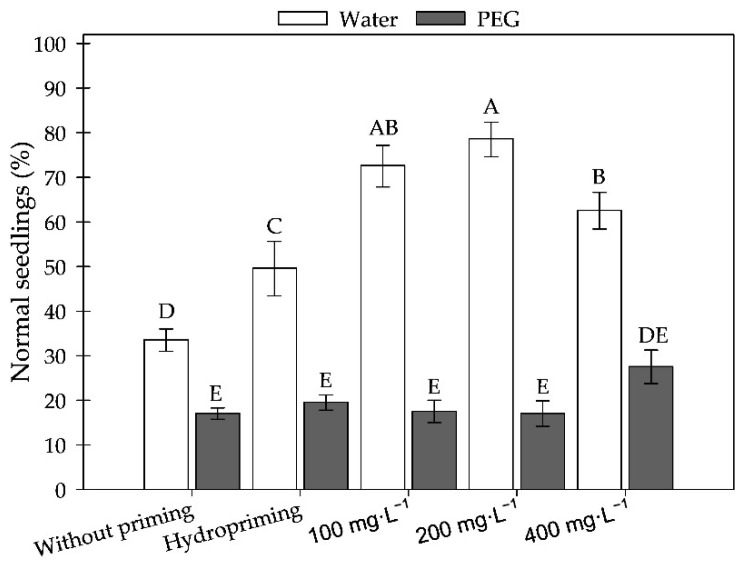
Normal sunflower seedlings (%) exposed to different concentrations of carbon nanotubes developed under water and water deficit conditions (PEG). Equal letters do not differ statistically from each other according to the Scott-Knott test (*p* ≥ 0.05).

Germination results did not differ among the treatments are therefore not presented. The percentage of normal seedlings was significantly reduced under water stress, induced by polyethylene glycol (PEG −0.6 MPa) compared to the control condition (H_2_O), highlighting the negative effects of water deficit on early development (Figure 8). The treatment without priming resulted in the lowest rates of normal seedlings. Although priming treatments supported seedling growth under H_2_O—particularly at concentrations of 100 and 200 mg·L^−1^ of MWCNTs—none were able to reverse the effects of stress under PEG conditions. Hydropriming showed an intermediate performance, whereas the 400 mg·L^−1^ concentration exhibited a slight reduction in efficiency. Thus, although germination rates were similar across treatments, the results indicate that nanopriming with 100 and 200 mg·L^−1^ may enhance early seedling development under non-stress conditions.

When using only water, the treatments with 100 and 200 mg·L^−1^ showed the best results, while the 400 mg·L^−1^ treatment exhibited a slight reduction. Hydropriming showed an intermediate performance. Thus, although germination was similar across treatments, PEG negatively affected the development of normal seedlings and was detrimental to all treatments. Conversely, priming with 100 and 200 mg·L^−1^ favored seedling growth under non-stress conditions.

**Figure 9:**
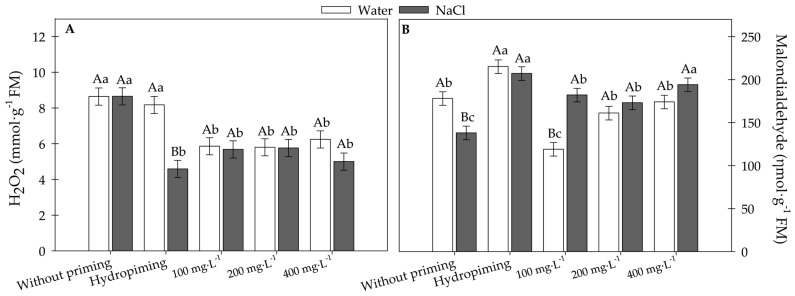
(**A**) Hydrogen peroxide and (**B**) lipid peroxidation in sunflower seedlings exposed to different concentrations of carbon nanotubes and germinated under saline conditions. Error bars represent the standard error (SE). Equal letters do not differ statistically from each other according to the Scott-Knott test (*p* ≥ 0.05). Uppercase letters compare germination conditions within the same carbon concentration, and lowercase letters compare the different carbon concentrations within the same germination condition.

Biochemical analyses were used to evaluate the impact of different priming treatments on hydrogen peroxide concentration and lipid peroxidation (Figure 9A,B) under H_2_O and NaCl conditions. Hydrogen peroxide (H_2_O_2_) levels were the highest in the no-priming treatment, with no significant difference between H_2_O and NaCl. Hydropriming significantly reduced the H_2_O_2_ concentration under NaCl stress but had no effect under H_2_O conditions. Treatments with 100, 200, and 400 mg·L^−1^ MWCNTs lowered H_2_O_2_ levels compared with no priming, with no significant differences between H_2_O and NaCl, suggesting that priming contributed to alleviating oxidative stress.

Lipid peroxidation was elevated in the no-priming treatment, showing lower values under NaCl than under H_2_O. Hydropriming maintained high malondialdehyde (MDA) levels, with no differences between H_2_O and NaCl. The 100 mg·L^−1^ treatment significantly reduced lipid peroxidation under H_2_O but remained elevated under NaCl. Treatments with 200 and 400 mg·L^−1^ exhibited intermediate MDA levels without significant differences between H_2_O and NaCl.

**Figure 10:**
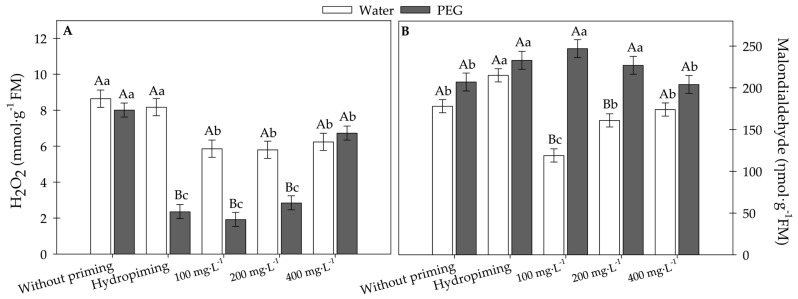
(**A**) Hydrogen peroxide and (**B**) lipid peroxidation in sunflower seedlings exposed to different concentrations of carbon nanotubes and germinated under water and water deficit conditions. Error bars represent the standard error (SE). Equal letters do not differ statistically from each other according to the Scott-Knott test (*p* ≥ 0.05). Uppercase letters compare germination conditions within the same carbon concentration, and lowercase letters compare the different carbon concentrations within the same germination condition.

Figure 10 shows that nanopriming with MWCNTs, particularly at concentrations of 100 and 200 mg·L^−1^, was effective in mitigating oxidative stress in sunflower seedlings under PEG-induced stress, significantly reducing H_2_O_2_ (Figure 10A) and MDA levels (Figure 10B).

The 100 mg·L^−1^ dose stood out for reducing lipid peroxidation under non-stress conditions, while the 400 mg·L^−1^ concentration did not provide the same benefits, suggesting that higher doses may compromise the treatment’s efficacy. These findings highlight the potential of MWCNTs as a promising tool for managing abiotic stresses, provided they are applied at appropriate concentrations.

## 3. Discussion

### 3.1. Accelerated Aging Experiment

Accelerated aging tests involve subjecting seeds to controlled stress conditions, such as high temperature and humidity, to evaluate their longevity and quality. Seeds with high vigor exhibit a greater capacity to tolerate these adverse conditions and tend to take longer to deteriorate [19,20]. Ref. [21] emphasized that, in addition to these factors, the accumulation of toxic metabolites is one of the main mechanisms driving seed aging, resulting in a significant reduction in germination rate, as observed after 72 h of accelerated aging.

MWCNTs have the ability to penetrate seeds, creating “nanoscale spaces” that facilitate the small-scale transport of water and nutrients through the cell wall and plasma membrane, either via ion channels or endocytosis [22]. Additionally, these nanotubes stimulate the expression of genes related to aquaporins, which facilitates water transport into cells [23]. The channels of carbon nanotubes are hydrophobic, extremely narrow, and stabilized by hydrogen bonds. These features enhance the movement of water molecules, increase the rate of water uptake, and promote plant growth and development [24].

The effectiveness of the MWCNTs was primarily observed after 48 h of accelerated aging. However, under prolonged aging periods, the carbon nanotubes were not effective in mitigating the damage caused by the accumulation of toxic metabolites under stress conditions. It was observed that, in the test conducted after 72 h, MWCNTs may have exerted a toxic effect on the plants, as all treatments involving nanotubes resulted in a lower percentage of normal seedlings compared to hydropriming within the same time frame.

The increased efficiency of water absorption and transport promoted by carbon nanotubes enhances the growth of both the root system and the shoot of seedlings. Ref. [25] reported that carbon nanotubes intensify enzymatic activity, thereby directly influencing plant growth. After 48 h of accelerated aging, a concentration of 200 mg·L^−1^ of MWCNTs was identified as optimal for improving these parameters, as also observed by [26] in *Clitoria ternatea* L. seedlings. These results highlight the beneficial potential of carbon nanotubes for plant development and their promising applications in agriculture.

A decrease in the a* values indicates a shift in the green spectrum toward yellow-red; however, the samples would only be classified within this new spectrum if the a* values were positive. The change in the green hue of the seedlings reflects the behavior of the foliar pigments. The increase in the a* values and decrease in the b* value in the presence of carbon nanotubes indicate subtle chlorophyll degradation accompanied by the synthesis of other pigments, such as carotenoids and xanthophylls [27]. This alteration resulted in seedlings with less green, more yellowish tones, and a slight bluish hue, which potentially reflect benefits for seedling metabolism, promoting greater resistance to abiotic stresses. Ref. [28] also reported a significant increase in the synthesis of various xanthophylls in rice seedlings treated with 200 mg·L^−1^ MWCNTs, reinforcing the influence of these nanomaterials on the modulation of plant pigment profiles.

The synthesis and degradation of pigments in response to carbon nanotubes vary depending on their concentration and plant species. In rice seedlings treated with 200 mg·L^−1^ MWCNTs, an increase in chlorophyll concentration was observed without significant changes in carotenoids [29]. In contrast, treatments with higher concentrations, such as 250 mg·L^−1^, led to increased production of β-carotene, which is responsible for yellow and orange tones, and chlorophyll b in tomato seedlings [30]. Conversely, studies in maize have shown that high concentrations of MWCNTs may induce the formation of reactive oxygen species (ROS), causing oxidative damage to chloroplasts and impairing chlorophyll production [31]. These findings highlight the importance of adjusting MWCNT concentrations according to species and intended agronomic application.

Ref. [32] suggested that high concentrations of MWCNTs can induce oxidative stress, as evidenced by the elevated H_2_O_2_ levels and moderate lipid peroxidation in samples exposed to the maximum nanotube concentration tested in this study. Furthermore, ROS also act as positive signaling molecules in the seed dormancy-breaking process [33]. In this context, treatments such as hydropriming, are expected to promote increased H_2_O_2_ production, intensify lipid peroxidation, and enhance the activity of reducing enzymes. This technique is particularly relevant as it stimulates the antioxidant system, thereby contributing to the improvement of seed physiological quality [34].

SOD, predominantly located in the mitochondria, is responsible for catalyzing the conversion of superoxide radicals into hydrogen peroxide and plays an essential role in plant tolerance to various types of stress [35]. In the present study, hydropriming significantly increased SOD activity compared to MWCNT treatments, indicating an active enzymatic antioxidant response under stress conditions. However, seeds primed with 200 mg·L^−1^ of MWCNTs, despite exhibiting lower SOD activity, showed reduced levels of hydrogen peroxide and lipid peroxidation, suggesting that alternative mechanisms, possibly involving non-enzymatic antioxidants or enhanced membrane stability, may be contributing to oxidative stress mitigation in this treatment.

In hydropriming treatments, the increase in hydrogen peroxide (H_2_O_2_) concentration and lipid peroxidation was accompanied by elevated activities of superoxide dismutase (SOD) and catalase (CAT), which mitigate the effects of oxidative stress. For maize, ref. [36] identified 200 mg·L^−1^ of MWCNTs as the optimal concentration for reducing SOD activity. Catalase, primarily located in peroxisomes, not only converts H_2_O_2_ into water and oxygen but also plays an important role in triggering plant senescence, during which antioxidant enzyme activities decrease to allow the accumulation of ROS [37]. Moreover, catalase is crucial in plant defense, contributing to immune system strengthening and mitigating damage caused by ROS generated during interactions with pathogenic microorganisms [38].

In millet seedlings, concentrations above 90 mg·L^−1^ MWCNTs triggered increased synthesis of peroxidase, CAT, and SOD [39]. In the present study, the increase in H_2_O_2_ observed in the hydropriming treatments (compared to non-primed seeds) was accompanied by increases in SOD and CAT activities as a response to oxidative stress. Thus, carbon nanotubes can also be considered regulators of antioxidant system enzymes [40]. In plants of the genus *Arabidopsis*, it has been shown that H_2_O_2_ accumulation can induce activation of calcium channels and stomatal closure to maintain cellular homeostasis [41].

The ascorbate–glutathione cycle plays a fundamental role in the antioxidant defense system of plants. In this cycle, ascorbate is catalyzed by ascorbate peroxidase (APX), which is converted into monodehydroascorbate (MDHA) while reducing H_2_O_2_ to H_2_O. In parallel, the glutathione cycle regenerates ascorbate, thereby maintaining the antioxidant system and reducing the oxidative activity of ROS [42].

In this study, when comparing the 200·mg L^−1^ carbon nanotube treatment with the negative control (no priming), a reduction in peroxide levels was observed without a corresponding increase in the enzymatic activities of SOD, CAT, or APX. These results suggest that carbon nanotubes act through alternative mechanisms to reduce the H_2_O_2_ levels. It is possible that MWCNTs interact directly with the phytochemical antioxidants such as betaine and allantoin, capable of neutralizing H_2_O_2_ [43].

### 3.2. Germination Stressful Conditions Experiment

The results of this study demonstrated that nanopriming seeds with multi-walled carbon nanotubes (MWCNTs), particularly at concentrations of 100 and 200 mg·L^−1^, significantly improved sunflower seedling performance under both control and stress conditions, notably salinity (NaCl).

The similar germination rates observed under water and saline conditions in MWCNT-treated seeds suggest the mitigating effect of nanopriming on salt stress. Previous studies have corroborated that carbon-based nanomaterials can enhance seed germination and seedling vigor by modifying water uptake kinetics and activating stress-response genes [44,45]. For instance, ref. [46] showed that carbon nanotubes can penetrate seed coats, enhance water absorption, and promote metabolic activity. Although hydropriming also improved seedling performance under saline conditions, MWCNT treatments were more effective in maintaining the percentage of normal seedlings. This is consistent with [47], who found that ZnO nanoparticles improved the salt tolerance of rice seedlings by upregulating the antioxidative system.

PEG-induced osmotic stress significantly reduced seedling development in all treatments, indicating more severe physiological impact compared to salt stress. The treatment with an MWCNT concentration of 400 mg·L^−1^ was the only condition showing some improvement in seedling devevolpment under water deficit stress (PEG). This can be explained by results found with the use of nanomaterials in plants, where it has been reported that MWCNTs have been associated with the activation of genes related to water transport, such as those encoding aquaporins (proteins responsible for transporting water molecules across cell membranes). The role of this protein is related to the enhancement of water uptake and transport, reducing the effect of abiotic stress [48].

Nevertheless, MWCNT priming (100 and 200 mg·L^−1^) still yielded better outcomes for normal seedlings developed under water conditions than hydropriming or no priming. This supports the findings of [25], who reported that CNTs can alleviate drought stress by promoting aquaporin expression and enhancing water transport efficiency.

H_2_O_2_ and lipid peroxidation were used as markers of oxidative stress. Both were significantly reduced by MWCNT treatments compared to non-primed controls. Notably, 100 and 200 mg·L^−1^ concentrations were the most effective, corroborating with [31], who observed that CNTs reduced oxidative stress in maize seedlings by enhancing antioxidant enzyme activities and stabilizing membrane integrity. On the other hand, 400 mg·L^−1^ of MWCNTs showed diminished benefits or slight toxicity, as indicated by elevated oxidative stress markers. This reflects the dose-dependent dual effect of nanomaterials, where lower concentrations are beneficial, but higher doses may disrupt the redox balance or lead to nanoparticle-induced toxicity [32,36].

Hydropriming moderately reduced oxidative stress under NaCl conditions but was less effective than nanopriming. These findings are in agreement with those reported by [34], demonstrating that nanopriming is among the most effective strategies to increase salt tolerance in plants, by improving both physiological performance and biochemical resilience.

Overall, nanopriming with MWCNTs at 100–200 mg·L^−1^ emerges as a promising strategy for improving sunflower seedling tolerance to salinity and, to a lesser extent, drought-like stress. The results underscore the potential of integrating nanotechnology into seed priming approaches, while also emphasizing the importance of concentration control to avoid phytotoxic effects.

The most immediate and practical application of these findings lies in the commercial seed treatment industry. Nanopriming, similar to existing seed conditioning methods such as hydropriming or osmopriming, would involve coating or soaking sunflower seeds in a carefully calibrated MWCNT solution prior to distribution and planting. This pre-sowing treatment ensures that the seeds arrive at the farm already fortified against early-stage abiotic stresses. Seed companies could develop specialized nanoprimed sunflower varieties, providing farmers with a “ready-to-plant” solution that inherently possesses greater stress tolerance. The low concentrations considered optimal (100–200 mg L^−1^) are economically attractive, suggesting that the cost of treatment can be justified by the improved performance and subsequent productivity benefits.

## 4. Materials and Methods

The experiment was conducted at the Central Laboratory for Seed Research in the Department of Agriculture at the Federal University of Lavras, using sunflower seeds (*Helianthus annuus*). Seeds were divided into two lots, totaling two experiments. In the first experiment, the seeds were subjected to accelerated aging, followed by conditioning with multi-walled carbon nanotubes (MWCNTs) at different concentrations. In the second lot, the seeds were conditioned with MWCNTs at different concentrations and then exposed to two stress conditions: salt stress and water deficit.

MWCNTs were provided by Center for Technology in Nanomaterials and Graphene (CTNano)—Federal University of Minas Gerais (UFMG—acronym in Portugues), Belo Horizonte, Brazil, with an average diameter of 19 nm, a length distribution of up to 30 μm, an average length of 6 μm by arithmetic mean, and 12 μm by weighted mean. They were produced by chemical vapor deposition, and functionalization used sulfuric acid (P.A. ACS FMaia 95.0–98.0%) and nitric acid (P.A. Anidrol minimum dosage 65%).

### 4.1. Accelerated Aging Experiment

The seeds were disinfected before starting the test to prevent the development of microorganisms. The seeds were initially immersed in a 1% sodium hypochlorite (NaClO) solution for 3 min. Then, after rapid drying on sterilized filter paper, the seeds were subjected to the accelerated aging test [49]. For the first lot, the accelerated aging test was conducted using the plastic box method [50], in which sunflower seeds were distributed in a single layer over a screen (200 g of seeds), and hermetically sealed to maintain constant moisture. The boxes were placed in a Biochemical Oxygen Demand (B.O.D.) chamber at 40 °C for 24, 48, and 72 h, corresponding to three treatments. After aging, the seeds were conditioned as described below.

For the physiological conditioning using MWCNTs, a stock solution was prepared using 1 g of carboxylic acid-functionalized MWCNTs, in 1 L of distilled water, resulting in a concentration of 1000 mg·L^−1^ MWCNTs. The solution was placed in a beaker and sonicated (Branson—Digital Sonifier^®^, models 250 & 450) at 50% amplitude for 15 min, repeated three times. After homogenization, the solution was transferred to an amber bottle, wrapped in aluminum foil, and refrigerated until use.

From the stock solution, dilutions in distilled water were made to obtain working solutions with concentrations of 100 mg·L^−1^, 200 mg·L^−1^, and 400 mg·L^−1^ MWCNTs. The criteria used to determine the concentrations of MWCNTs were based on reviewed literature and published articles in the field. Sunflower seeds were then immersed in aerated containers prepared for each concentration of MWCNTs, for 24 h at 15 °C in the dark. Seeds were also conditioned with distilled water for the hydropriming for 24 h at 15 °C in the dark. After priming, the seeds were dried in a forced-air circulation oven at 25 °C for 24 h. After this period, germination tests were performed, along with morphological and biochemical analyses of the seedlings, on seeds from all treatments, including control seeds (without accelerated aging time and priming treatment) as described below.

**Germination Test:** Seeds of all treatments were placed between sheets of germination paper, moistened with distilled water in an amount equivalent to 2.5 times the weight of the dry paper. After that, they were wrapped in paper towels, and the rolls were placed inside plastic bags to prevent moisture loss. Each roll contained 50 seeds with four replicates per treatment. The rolls were incubated in B.O.D. type chamber at a constant temperature of 25 °C with a 12 h photoperiod [51]. The experiment was performed for 14 days, during which the root protrusion and the percentage of normal seedlings were evaluated.

**Morphological parameters** were evaluated only for the best treatment identified in the accelerated aging experiment. Root and shoot lengths were measured on day 14 and resulteds presentd in centimeters. All normal seedlings were photographed and analyzed using AxioVision 4.8 software, which was calibrated according to a scale derived from the images and used to measure the morphological parameters assessed.

**Colorimetric Parameters:** Also, on day 14, and based on the best treatment from the accelerated aging experiment, sunflower seedlings exposed to different concentrations of MWCNTs were photographed and analyzed using Adobe software to evaluate the LAB colorimetric parameters of the cotyledon leaves. The measured parameters included: a* (dimensionless), representing the wavelength range for red (positive values) and green (negative values); b* (dimensionless), representing the wavelength range for yellow (positive values) and blue (negative values); and L* (dimensionless), representing the brightness of the sample (the more positive the value, the lighter the sample; the more negative, the darker) [52].

**Quantification of Hydrogen Peroxide and Lipid Peroxidation:** 200 mg samples of seedlings collected on day 14 were ground in liquid nitrogen with the addition of 20% PVPP (*w*/*v*), homogenized in 1.5 mL of 0.1% trichloroacetic acid (TCA, *w*/*v*), and centrifuged at 12,000× *g* for 15 min at 4 °C. Hydrogen peroxide levels were determined by measuring the absorbance at 390 nm in a reaction medium containing 100 mM potassium phosphate buffer (pH 7.0) and 1 M potassium iodide [53].

Lipid peroxidation was determined by quantifying thiobarbituric acid-reactive substances (TBARS) [54]. The extract was prepared following [53]. Aliquots (250 µL) of the supernatant were added to a reaction mixture containing 0.5% thiobarbituric acid (TBA, *w*/*v*), and 10% TCA (*w*/*v*), and then incubated at 95 °C for 30 min. The reaction was stopped by rapid cooling on ice, and absorbance readings were recorded at 535 nm and 600 nm using a spectrophotometer.

**Antioxidant Enzyme Activity:** Enzymes were extracted using 200 mg of fresh mass from normal sunflower seedlings in each treatment. The tissue was ground in liquid nitrogen and homogenized in 1.5 mL extraction buffer. The homogenates were centrifuged at 12,000× *g* for 30 min at 4 °C, and the supernatants were collected for enzymatic analysis, as described previously [55].

Superoxide dismutase (SOD) activity was determined based on the enzyme’s ability to inhibit the photochemical reduction in nitro blue tetrazolium (NBT), as proposed previously [56]. Tubes containing buffer along with the sample and the control (incubation medium without the sample) were illuminated with a 20 W fluorescent lamp for 7 min, and absorbance was measured at 560 nm. One unit of SOD activity is defined as the amount of enzyme required to inhibit 50% of the NBT reduction rate. The absorbance was measured at 560 nm using a spectrophotometer.

Catalase (CAT) activity was determined by the decrease in absorbance at 240 nm every 15 s for 3 min, reflecting the consumption of hydrogen peroxide (H_2_O_2_), as described previously [57]. The reaction was initiated by adding H_2_O_2_ (ε = 36 mM^−1^ cm^−1^). One unit of CAT is defined by the amount of enzyme required to decompose 1 µmol of H_2_O_2_ per minute.

Ascorbate peroxidase (APX) activity was determined by monitoring the decrease in ascorbate absorbance (ε = 2.8 mM^−1^ cm^−1^) at 290 nm every 15 s over 3 min, according to [58]. One unit of APX is defined by the amount of enzyme that oxidizes 1 µmol of ascorbic acid per minute.

### 4.2. Germination Stressful Conditions Experiment

Using the same methodology described in the first experiment, a second lot of seeds was divided into four treatments: seeds conditioned with 100 mg·L^−1^, 200 mg·L^−1^, and 400 mg·L^−1^ MWCNTs, and unconditioned seeds (control) [59]. After conditioning, the seeds were subjected to different germination conditions: control (distilled water), salt stress using NaCl solution (−0.45 MPa) and water deficit induced by polyethylene glycol 6000 (PEG −0.6 MPa) [60]. The seeds were evaluated by germination tests, along with morphological and biochemical analyses of the seedlings using the same methodology as described in the first experiment.

### 4.3. Experimental Design and Statistical Analyses

The experimental design of the first experiment followed a completely randomized design (CRD) in a 4 × 4 + 1 factorial scheme, corresponding to four levels of accelerated aging (0 h, 24 h, 48 h, and 72 h), four concentrations of multi-walled carbon nanotubes (MWCNTs) (0 mg·L^−1^—hydropriming, 100 mg·L^−1^, 200 mg·L^−1^, and 400 mg·L^−1^), and a negative control consisting of seeds without accelerated aging and without physiological conditioning.

The second experiment was a completely randomized design in a factorial scheme (4 × 2 + 1), testing four concentrations of MWCNTs (0 mg·L^−1^—hydropriming, 100 mg·L^−1^, 200 mg·L^−1^, and 400 mg·L^−1^) and two types of stress (NaCl and PEG), along with a negative control consisting of non-primed seeds under non-stress conditions (H_2_O).

All the data were subjected to analysis of variance (ANOVA) and the Scott-Knott test for mean comparisons at a 5% significance level, using SISVAR 5.8 statistical software [61]. Dunnett’s test was applied at a 5% probability level to compare treatment means against the control (without accelerated aging time and priming treatment).

## 5. Conclusions

This study reinforces the use of multi-walled carbon nanotube (MWCNT) nanopriming as an innovative and promising strategy for agriculture by demonstrating its beneficial effects on sunflower seedling development. Under controlled conditions, treatment with 200 mg·L^−1^ MWCNTs notably enhanced root growth, strengthened the aerial part, and positively modulated the antioxidant system, thereby reducing oxidative stress, especially under saline stress conditions.

Based on the results obtained in this study with MWCNTs, the application of this technology to seeds can optimize seed vigor under stress conditions, contributing to the development of improved agricultural formulations and driving more sustainable agriculture through greater crop resilience and productivity. However, theses results also highlight the need for caution, as higher concentrations may exacerbate oxidative stress. Therefore, despite the promising outcomes observed, further studies are necessary to elucidate the underlying mechanisms and ensure the safe and efficient application of this technology in agricultural practices.

## Data Availability

The datasets generated and/or analyzed during the current study are available from the corresponding author upon reasonable request.

## Abbreviations

The following abbreviations are used in this manuscript:

MWCNTs	Multi-walled carbon nanotubes
ROS	Reactive oxygen species
H_2_O_2_	Hydrogen peroxide
SOD	Superoxide dismutase
CAT	Catalase
APX	Ascorbate peroxidase
NaCl	Sodium chloride
PEG	Polyethylene glycol
H_2_O	Water
MDA	Malondialdehyde
MDHA	Monodehydroascorbate
